# Hippocampal volume and white matter impairments in patients with congenital adrenal hyperplasia

**DOI:** 10.1210/clinem/dgag312

**Published:** 2026-07-31

**Authors:** Alice Savoia, Diana Kancsar, Anna Nordenström, Annelies van’t Westeinde, Svetlana Lajic

**Affiliations:** Department of Women's and Children's Health, Karolinska Institutet, Pediatric Endocrinology Unit, Karolinska University Hospital, Stockholm, SE-171 76, Sweden; Department of Women's and Children's Health, Karolinska Institutet, Pediatric Endocrinology Unit, Karolinska University Hospital, Stockholm, SE-171 76, Sweden; Department of Women's and Children's Health, Karolinska Institutet, Pediatric Endocrinology Unit, Karolinska University Hospital, Stockholm, SE-171 76, Sweden; Department of Women's and Children's Health, Karolinska Institutet, Pediatric Endocrinology Unit, Karolinska University Hospital, Stockholm, SE-171 76, Sweden; Department of Clinical Sciences, Sahlgrenska Academy, University of Gothenburg, Gothenburg, SE-416 50, Sweden; Department of Women's and Children's Health, Karolinska Institutet, Pediatric Endocrinology Unit, Karolinska University Hospital, Stockholm, SE-171 76, Sweden

**Keywords:** CAH, hippocampal subfields, white matter integrity, working memory, resting-state functional connectivity

## Abstract

**Context:**

Patients with congenital adrenal hyperplasia (CAH) have lifelong imbalances in adrenal hormones that may result in changes in structure and function of the brain that can impair cognitive performance. The hippocampus might be especially sensitive to cortisol and androgen disturbances but has not been systematically investigated in this patient group.

**Objective:**

In this study, we assessed hippocampal subfield volumes, mean diffusivity, and resting-state functional connectivity along with memory performance in patients with CAH and healthy controls using structural magnetic resonance imaging.

**Design:**

Cohort study; patients were recruited and tested as part of the PREDEX study.

**Setting:**

Single research institute.

**Participants:**

Twenty-nine patients with CAH (20 females, 9 males) and 33 healthy controls (19 females, 14 males).

**Intervention(s):**

No intervention was performed.

**Main outcome measure(s):**

Hippocampal subfield volumes, mean diffusivity, resting-state functional connectivity, and memory performance.

**Results:**

Patients with CAH had reduced left CA4 and left and right subiculum volumes. In the group by sex interaction analyses, males presented higher mean diffusivity in left CA3 and CA4 compared to controls. When looking at males and females separately, it was identified that females had reduced volumes of the bilateral subiculum, CA1, and left CA3 and CA4. Hippocampal structural alterations were not associated with memory performance.

**Conclusions:**

Patients with CAH had structural alterations in several hippocampal subfield volumes, but there might be a discrepancy between males and females in sensitivity to white or gray matter impairments. The findings warrant further investigation into sex-specific differences in treatment sensitivity to optimize brain health in both men and women with CAH.

## Introduction

Congenital adrenal hyperplasia (CAH) is a group of autosomal recessive disorders with 21-hydroxylase deficiency (21OHD) being the most prevalent. This enzyme is necessary for the synthesis of cortisol and aldosterone in the adrenal gland. When cortisol production is insufficient, the reduced negative feedback on the hypothalamic–pituitary-adrenal axis leads to excessive adrenal androgen production and adrenal hyperplasia (1). In females with the classic forms of 21OHD, this can cause virilization of the external genitalia already in utero (1). The primary treatment for 21OHD is hormone replacement therapy, consisting of oral hydrocortisone 3 to 4 times per day and fludrocortisone once daily, which attempts to restore cortisol and androgen levels, suppresses excess ACTH (adrenocorticotropic hormone), and prevents adrenal androgen overproduction (1, 2). Although patients with CAH have low endogenous cortisol levels, long-term administration of glucocorticoids can produce atypical fluctuations in cortisol concentrations due to the difficulties of replicating the endogenous circadian and ultradian secretion of cortisol with the current treatment strategies.

Given that CAH involves lifelong dysregulation of both cortisol and adrenal androgens, such persistent hormonal imbalances may contribute to alterations in brain structure. An increased occurrence of alterations in white matter microstructure has been reported among CAH patients (3-6). Brain abnormalities are not confined to white matter only but have been observed in gray matter and brain volumes as well. Patients with CAH were shown to have less gray matter than healthy individuals in the amygdala, subiculum, the calcarine cortex, and the left lingual gyrus (7). In addition, patients have been found to have smaller brain volumes, reduced thickness in the rostral middle frontal gyrus, left superior parietal cortex, and right inferior parietal cortex (5), and smaller left intracranial volumes (8) and reduced hippocampal and amygdala volumes (8, 9). Hormonal imbalances in CAH may contribute to the observed brain structural changes, potentially also impacting cognitive development and performance. Previous studies found patients with CAH to have lower IQ (10, 11) and cognitive deficits in domains such as comprehension and pattern analysis (10), verbal and visuospatial working memory (12), interference/inhibition control (12), visual memory and free recall (13), short-term memory, spatial perception, and quantitative skills (14), as well as overall working memory performance (15). However, evidence on cognitive outcome in CAH patients remains mixed, as many studies report no significant differences from controls in general intelligence, executive functions, or memory (12, 14, 16-18), though inconsistencies may reflect CAH subtype, salt-wasting (SW) history, prenatal dexamethasone exposure, age, and timing of diagnosis, with better outcomes during childhood in those identified via neonatal screening.

The hippocampus, rich in glucocorticoid and mineralocorticoid receptors, is critical for memory and spatial navigation (19-21) and is particularly vulnerable to glucocorticoid excess, especially in the CA1 and CA3 regions (22-25). Chronic cortisol exposure in healthy adults is associated with reduced volumes of the CA1–3 subfields and the hippocampal head, as well as smaller overall hippocampal volumes, particularly in the right hemisphere (26, 27). The hippocampus also responds to estrogens and androgens and contributes to relational aspects of working memory (28, 29). In CAH patients, studies report reduced hippocampal volumes and altered white matter integrity, particularly in the subiculum and CA1, with higher glucocorticoid doses linked to lower working memory performance and higher mean diffusivity in these areas (8, 30). However, some studies found no hippocampal volume reduction (9), likely reflecting differences in methods, timing of diagnosis, or treatment practices.

The hippocampus, a region critical for memory and stress regulation, may therefore be particularly vulnerable to lifelong hormonal imbalances associated with CAH. In this study, we use a multimodal assessment combining structural MRI, diffusion tensor imaging (DTI), and resting-state functional connectivity that allows for a comprehensive evaluation of both anatomical integrity and functional networks, enabling us to examine how microstructural white matter alterations relate to connectivity patterns and cognitive performance. With this integrated approach, we aim to clarify the complex relationship between hormone-mediated brain changes and neurocognitive outcomes and to identify potential sex-specific vulnerabilities that may not be detectable using a single imaging modality.

## Materials and methods

### Participants

Patients were recruited from a study investigating somatic, metabolic, and neurocognitive outcomes in CAH and the effects of prenatal DEX (dexamethasone) treatment in the context of CAH (ie, the Swedish PREDEX study) (12, 31). Patients with CAH were enlisted from different regions of Sweden. All patients were diagnosed early through neonatal screening for 21-hydroxylase deficiency. Control participants were recruited from the Swedish Civil Registration System, living in the Stockholm County area, in the same age range, and with the same sex distribution as the patient group. Here, we focused on patients with CAH who had not received prenatal DEX treatment. The initial total sample consisted of 116 participants. After matching patients and controls by sex and age, the cohort comprised a total of 77 participants. After quality control steps, the final sample comprised 62 participants, namely 29 patients with CAH (20 females, 9 males) aged 16-32 years, and 33 healthy controls (19 females, 14 males) aged 19-29 years. Genotypes were grouped based on the severity of the mildest mutated CYP21A2 allele to create 6 groups for analyses. The groups were defined as A (*n* = *2*, I172N + R356W, SW (salt-wasting); *n* = *1*, R356W, SW), B (*n* = *8*, I2 splice, SW), C (*n* = *9*, I172N; *n* = *1*, R341W, SV (simple virilizing)), D (*n* = *5*, P30L, SV/NC (non-classic)), and E (*n* = *2*, V281L, NC). There were 11 patients with the salt-wasting form, 14 with the simple virilizing form, and 2 with the non-classic form. Four patients were treated with prednisolone and 15 with hydrocortisone. The conversion ratio used to determine hydrocortisone equivalents was (Prednisolone dose * 4)/Body surface.

All participants gave written informed consent to take part in the study. The study was approved by the regional ethical committee of Karolinska Institutet and by the Swedish Ethical Review Authority (DNR 99-153 [990517]; 030619; 2011/1764-32 [111201]; 140912; 2017/1658-32 [170831]; 2018/1037-32 [180518]; 2020-00564 [200414]).

### Data acquisition

The same image acquisition and preprocessing pipeline was followed as in previous publications (5, 32). MRI data were acquired on a 3-T scanner with an 8-channel head coil, including T1-weighted anatomical images, diffusion-weighted imaging, and fMRI during working-memory tasks and resting-state fMRI (rs-fMRI). For this study, analyses focused on the 8-minute eyes-closed rs-fMRI (TR = 2000ms, TE = 30 ms, voxel size 3 mm^3^) and high-resolution T1-weighted anatomical images (voxel size 1 mm^3^). White matter microstructure was assessed using FSL (FMRIB Software Library) tract-based spatial statistics (TBSS) (33) based on diffusion-weighted scans (TR = 7.4 s, 62 slices, voxel size: 2.3 × 2.3 × 2.3 mm, 60 directions diffusion-weighted images [*b* = 1500 s/mm^2^], and 8 images with no diffusion sensitization [*b* = 0 s/mm^2^]). During the rs-fMRI, participants were instructed to keep their eyes closed, stay awake, and let their thoughts wander.

### Segmentation of the hippocampus

Anatomical images were processed with FreeSurfer (recon-all) v7.4.1 (34) to reconstruct cortical and subcortical structures, which were manually inspected for errors in the gray-white and gray-pial surface boundaries and corrected if needed. From the participants with sufficient data quality from the initial FreeSurfer surface generation, hippocampal subfields were calculated based on an automated segmentation implemented in FreeSurfer v7.4.1 (35), which labels each voxel of the T1 MR images to one of the 40 predefined structures by using probabilistic brain atlases (35). The latest FreeSurfer version showed good scan-rescan reliability (36, 37). Using this automated tool, the subsequent subfields were produced: the hippocampal tail, subiculum-body, CA1-body, subiculum-head, hippocampal-fissure, presubiculum-head, CA1-head, presubiculum-body, parasubiculum, molecular layer HP-head, molecular layer HP-body, GC-ML-DG-head, CA3-body, GC-ML-DG-body, CA4-head, CA4-body, fimbria, CA3-head, HATA (hippocampus–amygdala transition area**)**, whole hippocampal body, whole hippocampal head, and whole hippocampus. We focused on the hippocampal subfields CA1, CA3, CA4, and subiculum (for a reference, see Fig. 1) because these regions constitute the core hippocampal circuitry most reliably segmented with current MRI methods. These subfields also contain glucocorticoid receptors and are therefore particularly sensitive to the atypical prenatal and postnatal steroid hormone environments characteristic of CAH. For each subfield, the extracted measures from the head and body were then summed to obtain a single value per subfield. This procedure was applied both to volumetric measures and to mean diffusivity values, providing the combined data used in subsequent statistical analyses.

**Figure 1:**
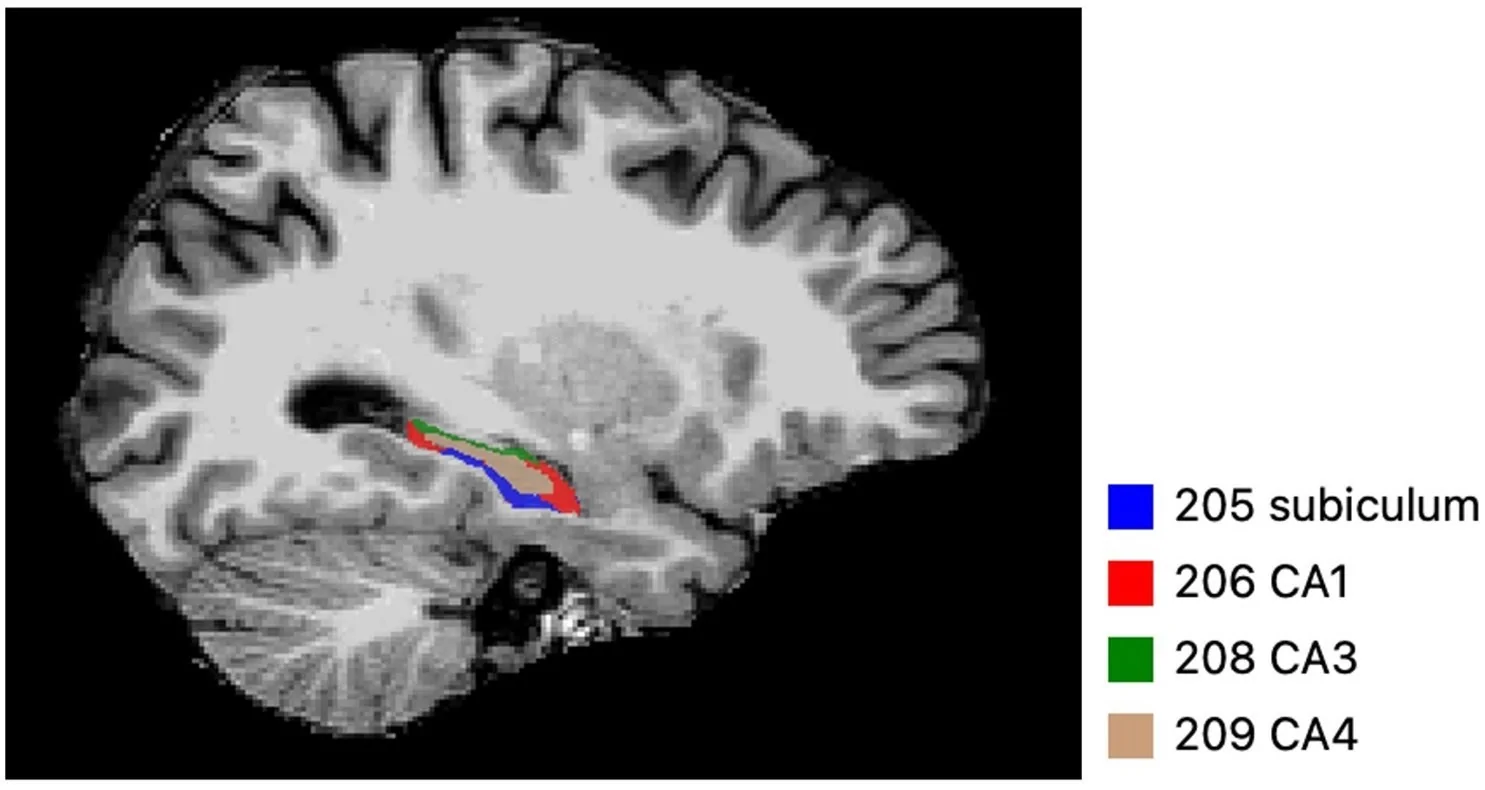
Right hippocampal subfield segmentation performed using FreeSurfer. Right hippocampal subfields included in the analyses are displayed as color-coded labels from the FreeSurfer look-up table overlaid on the native structural MRI of a representative participant. Abbreviation: CA, cornu ammonis.

### Analysis of diffusion tensor imaging data

Voxel-wise whole-brain TBSS analysis was run to obtain estimates of fractional anisotropy, mean diffusivity (MD), axial diffusivity, and radial diffusivity. Raw DTI data were corrected for eddy-current-induced distortions and head motion using the “*eddy*” tool provided by FSL (the FMRIB Software Library). The images were then corrected for susceptibility-induced distortions by registering the diffusion-weighted images to the non-diffusion-weighted images. A brain extraction tool was used to remove non-brain tissue from the DTI images.

Binary masks for hippocampal subfields were generated for each participant using anatomical MRI and diffusion-weighted images. Hippocampal subfield labels generated in FreeSurfer space were binarized using *mri binarize* and converted from MGZ to NIfTI format using *mri convert*.

Anatomical subfield masks were transformed into individual diffusion space using FSL FLIRT with the str2diff.mat transformation matrix. Transformed masks were re-binarized using *fslmaths* to ensure strict binary representation. These masks were used as seed regions for FSL tractography, generating subfield-specific tracts. Tract masks were then aligned with TBSS-derived MD images, and MD values were extracted for each subfield-specific tract. In the analysis, we focused on mean diffusivity only, which reflects the average rate of water diffusion, with higher values indicating greater white matter impairment.

### Functional connectivity analysis

#### Resting-state fMRI—preprocessing

Resting-state functional MRI data were preprocessed using FMRIB's Software Libraries version 5.0.11 (FMRIB Laboratory, University of Oxford) following a procedure described elsewhere (32).

#### Resting-state fMRI time series extraction

Resting-state fMRI time series were extracted from individual hippocampal subfields using a multistep registration and extraction pipeline implemented with FSL. Masks of hippocampal subfields were first created in diffusion space for each participant. These masks were then registered to the participants' high-resolution structural T1-weighted space using FSL's applywarp function with the subject-specific diff2str transformation matrix. Subsequently, the masks were transformed from T1 space into the individual functional space using a second registration step with the *highres2example func* matrix, aligning them with the rs-fMRI data. Following registration, the masks were binarized using FSL's *fslmaths* tool to create clean, binary regions of interest (ROIs) in functional space. Time series were then extracted from the preprocessed and denoised resting-state functional MRI data using FSL's *fslmeants* utility. This was done by applying the binarized masks as ROIs to the nuisance-regressed and motion-denoised functional data.

For each participant and each hippocampal subfield, a time series was generated, representing the mean BOLD (blood-oxygen-level-dependent-signal) signal within the ROI over time. This procedure was applied separately for left and right hemisphere subfields across participant groups: individuals with CAH and healthy controls. For CAH and controls, group-specific paths and transformation matrices were used to account for differences in acquisition parameters and preprocessing pipelines. All outputs, including registered masks and extracted time series, were saved for subsequent functional connectivity analyses.

#### First- and higher-level fMRI analysis

First-level statistical analyses were performed using FSL's FEAT (FMRI Expert Analysis Tool) to identify brain regions exhibiting activity correlated with the mean time series extracted from the hippocampal subfields, including the subiculum, CA1, CA3, and CA4 regions, in both the left and right hemispheres. For each subject, the respective design files (*design.fsf*) were used, corresponding to their group (CAH and control). The preprocessed and denoised functional data (*denoised func data nonaggr.nii.gz)* served as the 4D input data for the first-level model. Additionally, the corresponding brain-extracted structural image (*3D brain*) was provided as the 3D input for registration and normalization steps within FEAT. Following first-level analyses, higher-level analyses were performed to enable comparisons between subjects and groups.

### Neuropsychological assessment

Participants performed cognitive tests and filled in self-rating questionnaires (5). The neuropsychological tests, performed by a trained psychologist, included tests of verbal and nonverbal intelligence (WAIS-IV Vocabulary and WAIS-IV Matrices (38)), verbal and visuospatial working memory performance (WAIS-IV Digit Span (39) and Span Board Test (38)), learning and memory (WMS-III List Learning Test (39)), processing speed and interference (WMS-III Coding (38)), and the Stroop Task (40). For this study, we focused on verbal and visuospatial working memory performance and long-term memory in relation to hippocampal structure and function. In the present study, the term long-term memory is used to refer to declarative memory.

### Statistical analysis

All analyses were conducted in R 4.4.1 with significance set at *P* < .05. Continuous variables were standardized (z-scores).

#### Demographics

Group differences in demographic and clinical characteristics were assessed using one-way ANOVAs for continuous variables (eg, age) and chi-square tests for categorical variables (eg, education level), with a *P* value threshold of .05 for significant findings.

#### Group comparisons for brain morphology

Group comparisons between CAH and controls were performed for the estimate of white matter microstructure, namely MD, and hippocampal subfield volumes. We used ANCOVAs following the same steps: (1) whole-group comparison, (2) interaction between group (CAH and controls) and sex, (3) post hoc analyses splitting the group by sex for those tests where the interaction term was significant to also account for sex-specific effects, and (4) analysis split by sex. Results were considered significant with a *P* value of <.05. Covariates for whole-group comparison were age, sex, education level, and ICV (intracranial volume) (for volumes only). Covariates for interaction between group and sex were age, education level, and ICV (for volumes only).

#### Group comparisons for cognitive performance

Group comparisons between CAH and controls were performed for the estimates of verbal working memory, forward visuospatial working memory, backward visuospatial working memory, immediate recall, and long-term memory. We used ANCOVAs to investigate whole-group comparisons. Results were considered significant with a *P* value of <.05. Covariates were age, sex, and education level.

#### Associations between cognitive performance and brain morphology

We investigated whether brain morphology, namely hippocampal volumes and mean diffusivity values, could predict memory performance. Linear regression analyses were used. Significant effects were further explored with post hoc regression analyses stratified by group. False discovery rate (FDR) correction was applied to account for multiple comparisons in these analyses.

#### Associations between medication dose and brain morphology

Within sex-specific patient subgroups, we used linear regressions to predict hippocampal volumes and mean diffusivity values with the participants’ GC (glucocorticoid) dosage (hydrocortisone equivalents in mg/m^2^ body surface around the time of scanning, in the CAH group only).

#### Associations between genotype and brain morphology

Within the CAH group, we used ANCOVAs to compare hippocampal subfield volumes and mean diffusivity values across genotype groups with age and education level and ICV (for volumes only) as covariates. We followed these steps: (1) whole-group comparison and (2) post hoc analyses splitting by genotype group for those tests where the genotype term was significant. Results were considered significant with a *P* value of <.05

#### Resting-state analyses

Resting-state functional connectivity analyses within the hippocampus were conducted using seed-based correlation analyses, and group differences were tested using ANCOVAs with sex and age as covariates.

## Results

### Demographics

Congenital adrenal hyperplasia patients (23.16 ± 4.70 years) and controls (23.20 ± 3.14 years) had comparable mean ages (F (1,60) = .002; *P* = .966) and did not differ on other background variables (see Table 1).

**Table 1:** Demographic characteristics of the study population

Demographics	Females		Males		Test statistic (CAH vs controls)	*P* value
	CAH	Controls	CAH	Controls	/	/
*N*	20	19	9	14	/	/
Age (years)	23.7 ± 4.76	23.0 ± 3.26	21.9 ± 4.57	23.4 ± 3.10	F (1,60) = .002	.966
Education (≥ 3 years of university) (%)	26.3%	5.2%	22.2%	57.1%	X-squared = 3.8979e-31, df = 1	1
Medication dose GC (mg/m^2^)	12.3 ± 3.8	/	16.2 ± 5.6	/	/	/

Notes. Data are shown as mean ± SD for age and medication dose and counts for sex and education. Group differences were assessed using ANOVA for age and chi-square tests for education. Abbreviation: CAH, congenital adrenal hyperplasia.

### Effects of congenital adrenal hyperplasia on hippocampal volume and white matter microstructure

Patients with CAH had a smaller left CA4 (F (1,55) = 7.049, *P* = .010), left subiculum (F (1,55) = 4.063, *P* = .048), and right subiculum (F (1,55) = 4.53, *P* = .037) (Fig. 2A-2C and Table 2). No significant differences were found between patients with CAH and healthy controls for mean diffusivity of white matter (see Table 3).

**Figure 2:**
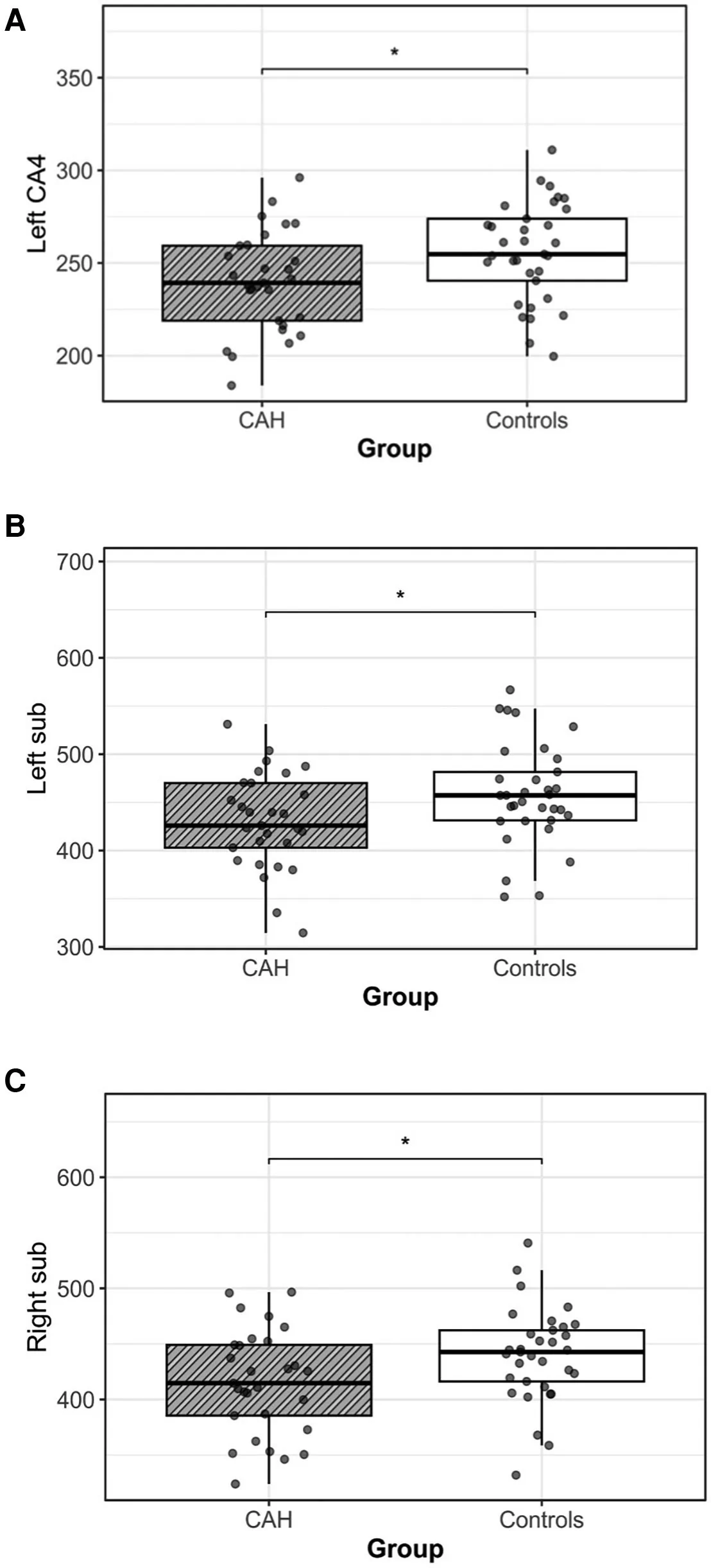
Left CA4 (A), left subiculum (B), and right subiculum (C) volumes in patients with CAH and healthy controls. Boxplots display the distribution of raw hippocampal subfield volumes for each group. The central line represents the median, the boxes represent the interquartile range, the whiskers indicate the minimum and maximum values, and individual participants are shown as overlaid dots. Abbreviations: CAH, congenital adrenal hyperplasia; CA, cornu ammonis; Sub, subiculum.

**Table 2:** Hippocampal subfield volumes in patients with CAH and healthy controls

Subfields	CAH	Controls	CAH vs controls	
			F (1,55)	*P*-value
ICV	1 461 435.87 ± 149 196.95	1 556 650.33 ± 151 499.09		
**Left subiculum**	**430.38** ± **49.70**	**458.27** ± **52.67**	**4**.**06**	.**048**
Left CA1	634.55 ± 93.43	669.31 ± 76.97	2.89	.09
Left CA3	199.18 ± 36.22	212.37 ± 30.91	2.98	.089
**Left CA4**	**239.94** ± **26.94**	**255.92** ± **26.68**	**7**.**04**	.**010**
**Right subiculum**	**415.40** ± **47.01**	**439.49** ± **42.52**	**4**.**53**	.**037**
Right CA1	673.58 ± 82.33	704.85 ± 74.35	2.49	.119
Right CA3	229.94 ± 35.81	231.07 ± 33.14	.003	.950
Right CA4	254.65 ± 29.29	262.47 ± 27.75	1.19	.279

Notes. Patients with CAH, compared to controls, had smaller left and right subiculum and left CA4. Mean volumes (±SD) are reported for each group. Group differences were assessed using ANCOVAs with age, sex, education, and intracranial volume as covariates. F and *P* values are reported for the main effect of diagnosis. Significant group differences are highlighted in **bold**. Abbreviations: CA, cornu ammonis; CAH, congenital adrenal hyperplasia; ICV, intracranial volume.

**Table 3:** Mean diffusivity values (mm^2^/s) in patients with CAH and healthy controls

Mean diffusivity	CAH (×10^3^)	Controls (×10^3^)	CAH vs controls	
			F (1,55)	*P*-value
Left subiculum	1.78 ± .07	1.75 ± .06	3.13	.082
Left CA1	1.97 ± .11	1.94 ± .11	.83	.364
Left CA3	2.01 ± .12	1.98 ± .16	.64	.425
Left CA4	2.00 ± .11	1.95 ± .12	2.17	.145
Right subiculum	1.80 ± .05	1.79 ± .06	.11	.737
Right CA1	1.93 ± .10	1.95 ± .15	.17	.673
Right CA3	2.04 ± .12	2.04 ± .20	.01	.901
Right CA4	2.02 ± .10	1.98 ± .15	1.37	.246

Notes. No significant difference was found between patients with CAH and controls. Mean MD values (±SD) are reported for each group. Group differences were assessed using ANCOVAs with age, sex, and education as covariates. F and *P* values are reported for the main effect of diagnosis. Abbreviations: CA, cornu ammonis; CAH, congenital adrenal hyperplasia.

### Effects of congenital adrenal hyperplasia on cognition

Patients with CAH obtained lower scores compared to healthy controls in the forward visuospatial working memory task (WMS-III, Span Board forward) (F (1,5) = 8.59, *P* = .004) (see Fig. 3 and Table 4).

**Figure 3:**
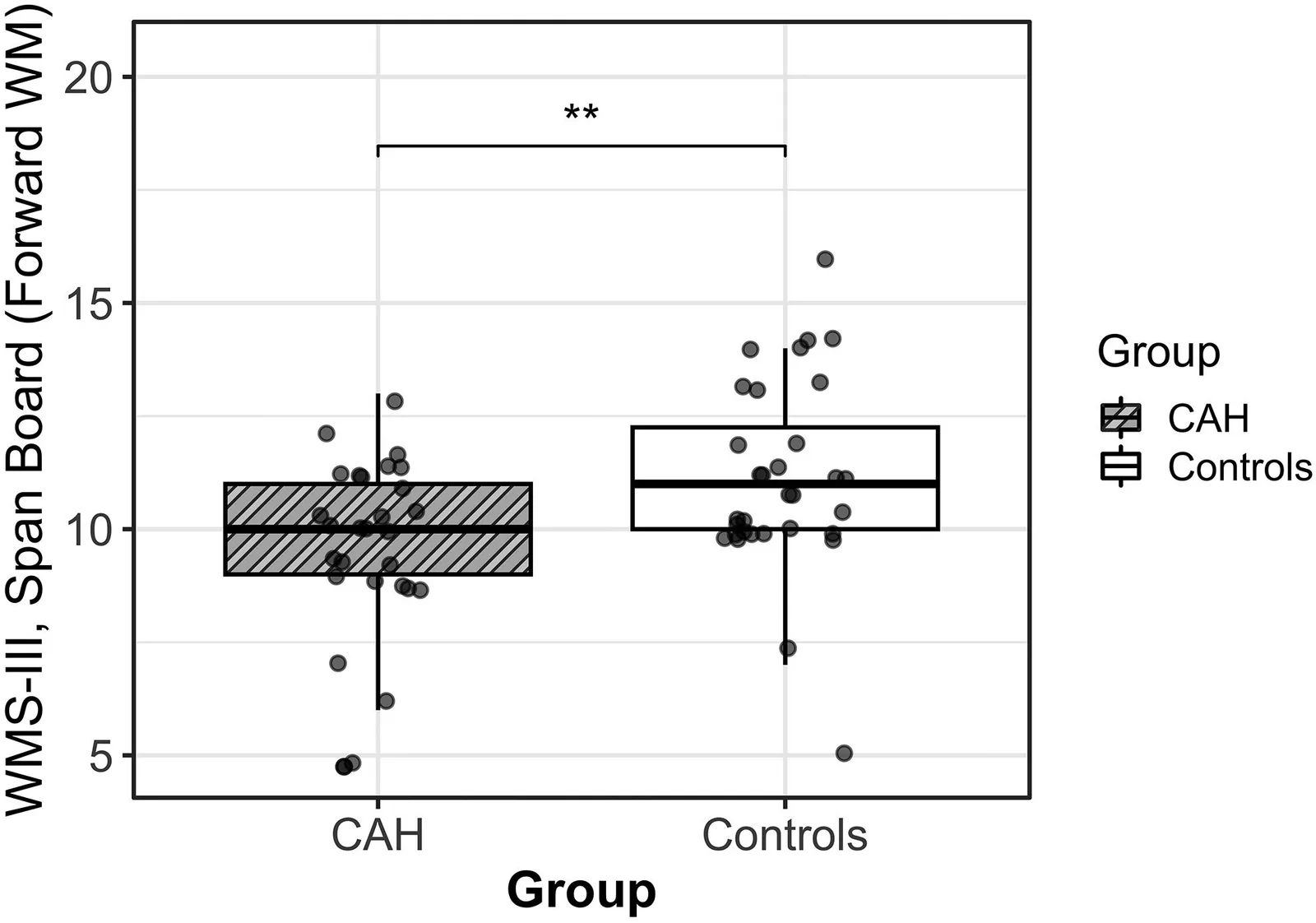
Visuospatial working memory performance in patients with CAH and healthy controls. Boxplots display Span Board forward scaled scores from the Wechsler Memory Scale for each group. The central line represents the median, the boxes represent the interquartile range, the whiskers indicate the minimum and maximum values, and individual participants are shown as overlaid dots. Abbreviations: CAH, congenital adrenal hyperplasia; WMS, Wechsler Memory Scale.

**Table 4:** Cognitive performance in patients with CAH and healthy controls

Cognitive measure	CAH (mean ± SD)	Controls (mean ± SD)	CAH vs controls	
			F (1,55)	*P* value
WAIS-IV, Digit Span (verbal WM) (S)	9.72 ± 2.56	10.28 ± 2.20	.95	.332
**WMS-III, Span Board (forward visuospatial WM) (S)**	**9.41** ± **2.07**	**11.06** ± **2.12**	**8**.**59**	.**004**
WMS-III, Span Board (backward visuospatial WM) (S)	11.20 ± 2.45	11.96 ± 1.5	2.20	.142
WMS-III, word list (Immediate recall) (S)	11.32 ± 3.42	10.81 ± 2.64	.17	.677
WMS-III, word list (LTM) (S)	13.39 ± 2.43	12.75 ± 2.12	1.47	.230

Notes. Mean scores (± SD) are reported for each group. Group differences were assessed using ANCOVAs with age, sex, and education as covariates. F- and *P*-values refer to the main effect of diagnosis. Significant group differences are highlighted in bold. Abbreviations: LTM, long-term memory; S, scaled scores; SD, standard deviation; WAIS, Wechsler Adult Intelligence Scale; WM, working memory; WMS, Wechsler Memory Scale.

### Association between hippocampal subfield volumes, white matter, and cognition

Within the CAH group, there was a significant association between the left CA4 and forward visuospatial working memory (β = −.53; *P* = .023), as a bigger left CA4 predicted a lower performance in the task. Moreover, there was a significant association between the right subiculum and immediate recall (β = .5; *P* = .033), as a bigger right subiculum predicted a better immediate recall performance. However, these associations did not survive FDR correction (*P* > .05).

### Sex-specific effects on hippocampal subfield volumes and white matter

There was a significant interaction between sex and diagnosis for the mean diffusivity in the left CA3 (F (1, 54) = 4.034, *P* = .049). Post hoc tests showed that males had higher MD in the left CA3 (*t*(54) = 2.06*, P* = .044, d = .559) and left CA4 (t(54) = 2.39, *P* = .020, d = .652) while females did not (*P* > .05). When analyses were conducted for males and females separately, it was again found that males with CAH, compared to control males, had higher mean diffusivity in the left CA3 (F (1,19) = 4.82, *P* = .040, η^2^p = .202) (Fig. 4A) and left CA4 (F (1,19) = 7.94; *P* = .010, η^2^p = .294) (Fig. 4B). We did not find any difference between patients and controls in females in white matter.

**Figure 4:**
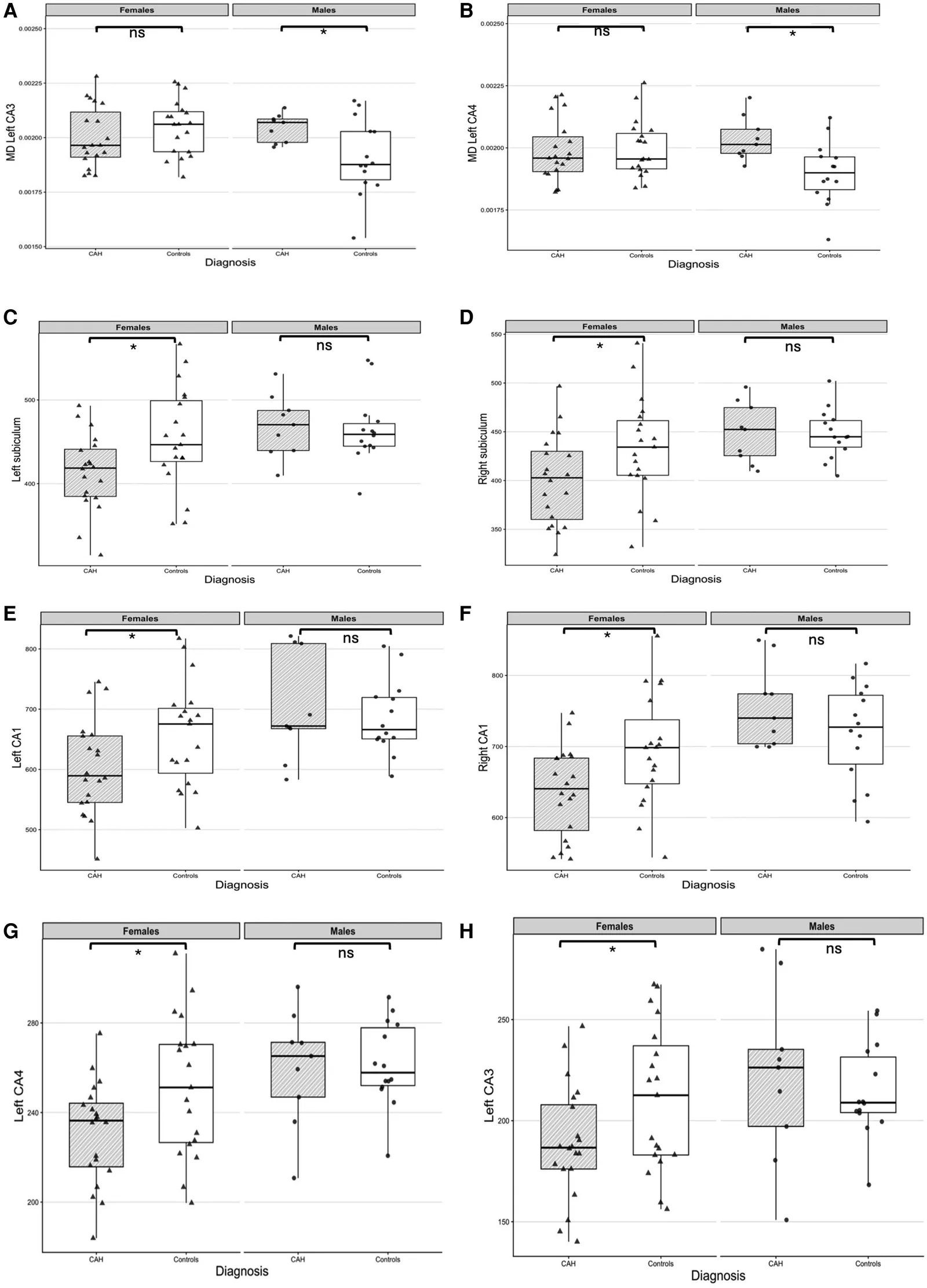
Mean diffusivity and hippocampal subfield volumes in women and men with CAH and healthy controls. Individual participant values are displayed as jittered points overlaid on boxplots showing the median and interquartile range. Female and male participants are presented separately, with patients with CAH and healthy controls shown side by side. Statistical comparisons were performed separately within each sex using linear models adjusted for age and education, followed by post hoc contrasts. Abbreviations: CAH, congenital adrenal hyperplasia; MD, mean diffusivity; CA, cornu ammonis; ns, not significant.

The interaction term for diagnosis and sex was not significant for any subfield volume. However, when comparing patients to controls within each sex group separately, we found that females with CAH, compared to control females, had smaller volumes of the left (F (1,33) = 5.15, *P* = .029, η^2^p = .135) and right (F (1,33) = 4.45, *P* = .042, η^2^p = .118) subiculum, left (F (1,33) = 4.401, *P* = .043, η^2^p = .117) and right (F (1,33)= 7.05, *P* = .042, η^2^p = .176) CA1, left CA4 (F (1,33)= 7.25, *P* = .011, η^2^p = .180), and left CA3 (F (1,33) = 5.495, *P* = .025, η^2^p = .142) (see Fig. 4C-4H). We did not find any difference between patients and controls in males.

### Associations between glucocorticoid dose and hippocampal subfield volumes and white matter in congenital adrenal hyperplasia patients

In CAH males, a higher GC dose was associated with a smaller left subiculum (β = −.5; *P* = .039), but no significant association was found with mean diffusivity. In CAH females, a higher GC dose was associated with lower mean diffusivity in the right CA1 (β = −.52; *P* = .019), but no significant association was found with volumes. However, these associations did not survive FDR correction.

### Associations between genotype and hippocampal subfield volumes and white matter in congenital adrenal hyperplasia patients

No significant associations with genotypes were found.

### Resting-state functional connectivity

Resting-state connectivity between hippocampal subfields and other brain areas did not reveal any significant differences between groups.

## Discussion

The present study assessed hippocampal subfield volumes, MD, and resting-state functional connectivity in the hippocampus related to working memory and long-term memory in patients with CAH.

Overall, patients with CAH had a smaller left CA4 and left and right subiculum compared to healthy controls, and male patients specifically had impairments in white matter in the left CA3 and CA4, and females had reduced volumes of the subiculum bilaterally, CA1 bilaterally, and left CA4 and CA3.

Despite the observation that the left CA4 and bilateral subiculum were smaller in patients with CAH, this alteration was not associated with memory problems in patients. Congenital adrenal hyperplasia patients in our sample did not have problems with long-term memory, a function which is typically associated with the hippocampus (41). Patients with a bigger right subiculum showed a better performance in the immediate recall task. However, patients did have problems with forward visuospatial working memory, but counterintuitively, patients with worse working memory performance had larger CA4 volumes. Since the hippocampus is not directly involved in working memory, this association can reflect other complex compensation mechanisms in other brain regions and networks. Indeed, working memory scores were in fact associated with cortical thinning of the middle frontal gyrus in this sample, as reported in an earlier publication (5). Working memory relies on a broader circuitry that extends beyond the hippocampus, and that critically involves the frontal regions. It is possible that memory encoding within the main hippocampal subfield regions (CA1-CA4) remains relatively preserved, while the transmission to other cortical regions, such as the prefrontal cortex, is less efficient.

In general, the lack of significant interaction effects with sex, apart from MD in left CA3, seemed to suggest that gross structural differences between the CAH and control group are similar in males and females. At the same time, even without a significant interaction term, when assessing hippocampal structure within the sexes separately, we found that white matter was impaired only in male patients, while gray matter volumes were impaired only in female patients. The lack of a significant interaction term can be due to increased variation in one sex as opposed to the other, meaning that the effect exists in one sex but is not convincingly enough absent in the other sex, ie, it could exist in only a subset.

Sex-specific differences in white matter microstructure may reflect differential sensitivity to hormonal influences, particularly glucocorticoids, which are known to affect myelination (42, 43).

These results are consistent with previous authors (5), who, in the same cohort, found that males with CAH exhibited higher mean axial diffusivity than females in the left forceps and theleft inferior fronto-occipital fasciculus. Moreover, diffusion studies in healthy population indicate that mean diffusivity can be higher in males compared to females in region-specific white matter tracts. For example, in children and adolescents, males exhibited significantly higher mean diffusivity across fronto-parietal-temporal regions compared to females (44, 45). Moreover, in studies of normal aging, males demonstrated a steeper increase in mean diffusivity in frontal, temporal, and parietal white matter (45). These findings indicate that males might be more sensitive to white matter microstructure alterations. Another contributing factor may be sex differences in treatment adherence, as males might exhibit more intermittent medication use, potentially exacerbating white matter damage.

Regarding gray matter volumes, females with CAH showed, compared to control females, smaller left and right subiculum, left and right CA1, and left CA4 and CA3. These effects were associated with medium-to-large effect sizes, suggesting that in females the diagnosis accounted for a substantial proportion of variance within this sex group. Congenital adrenal hyperplasia might be associated with differences in hippocampal subfield volumes, and sex-stratified analyses suggest that differences may be more apparent in females, although no significant interaction between sex and diagnosis was found.

Moreover, hippocampal structure was differentially associated with glucocorticoid replacement dose in male and female patients. A higher GC dose correlated with more impaired hippocampal structure in males, specifically lower volume of the left subiculum, while in females, a higher GC dose correlated with lower MD in the right CA1. Higher GC dose in females might have a protective effect on hippocampal white matter microstructure, potentially contributing to the lower mean diffusivity observed, whereas males may be more vulnerable to GC-linked volume reductions. In animal models (46), it was found that, following chronic corticosterone exposure, males showed dendritic atrophy in CA3 neurons while females showed minimal structural changes. Hippocampal structure therefore seems to be affected in a sex-specific manner in CAH, while the relationship with memory performance appears to be more complex.

Overall, it seems that the hippocampus is affected only to some extent in our sample of adolescents and young adults with CAH, who have all been treated from birth. In another cohort of adult females with CAH in the United Kingdom, the authors reported more widely distributed aberrations with smaller right hippocampal volume and increased mean diffusivity bilaterally in the hippocampus and other brain areas such as the superior and inferior longitudinal fasciculus, inferior fronto-occipital fasciculus, anterior thalamic radiation, corticospinal tract, uncinate fasciculus, and the cingulate gyrus (30). In contrast, an MRI study in children in the United States did not find robust hippocampal differences after correcting for total brain volume, suggesting that hippocampus involvement might vary depending on age, postnatal screening practices, treatment exposure, and cohort characteristics (5-8). Since we did not observe differences in resting-state connectivity in the hippocampus, the underlying alteration may be primarily structural. This also corresponds to findings from resting-state and working-memory task-related activity in our sample, where only minimal differences in functional activation were found between CAH patients and controls (5, 18).

This study has some limitations. First, our sample size was relatively small, particularly for assessing sex differences, and a larger sample could potentially reveal additional patterns. Second, since our study was cross-sectional, we cannot make strong conclusions about causality. It would be helpful to conduct longitudinal studies to see how these relationships develop over time. Additionally, it would be interesting to explore the accumulated levels of glucocorticoid exposure (medication doses) received by the patients over time, as this could significantly have an impact on their cognitive performance and brain structure. Moreover, another limitation of the present study is that handedness could not be adequately accounted for in the analyses due to the very small number of left-handed participants (*n* = 4). While handedness may be relevant in studies of hemispheric lateralization, the present sample size did not allow for a reliable statistical assessment of its potential effects.

The study emphasizes the complexity of how cortisol and adrenal hormonal imbalance can influence brain structure and brain function. Even if the hippocampus, which is a region critical for memory, did not show substantial volumetric differences in this relatively young patient cohort, the functional implications of white matter changes require further investigation. Given these findings, future research should use longitudinal studies to better understand how structural changes evolve over time and how these changes might correlate with memory performance in patients with CAH.

## Data Availability

Original data generated and analyzed during this study are included in this published article or in the data repositories listed in References.
